# Postbiotics Against Breast Cancer: A Narrative Review Bridging Preclinical Evidence with Potential Clinical Application

**DOI:** 10.3390/cancers18152486

**Published:** 2026-08-03

**Authors:** Chiara Luongo, Roberta Di Santillo, Alessia Cadavere, Franca Oglio, Laura Pisapia, Alessia Gaeta, Chiara Scocco, Juan Luis López-Cánovas, Marco Michelini, Monia De Aloe, Anna Lintura, Saranya Chumsri, Roberto Berni Canani

**Affiliations:** 1 Department of Translational Medical Science, University of Naples Federico II, 80138 Naples, Italy; luongoc@ceinge.unina.it (C.L.); disantillo@ceinge.unina.it (R.D.S.); cadavere@ceinge.unina.it (A.C.); oglio@ceinge.unina.it (F.O.); michelini@ceinge.unina.it (M.M.); dealoe@ceinge.unina.it (M.D.A.); lintura@ceinge.unina.it (A.L.); 2 NutriTechLab, CEINGE Advanced Biotechnologies Research Center, University of Naples Federico II, 80138 Naples, Italy; 3 ImmunoNutritionLab, CEINGE Advanced Biotechnologies Research Center, University of Naples Federico II, 80138 Naples, Italy; 4 Institute of Genetics and Biophysics, National Research Council (CNR), 80125 Naples, Italy; laura.pisapia@igb.cnr.it (L.P.); alessia.gaeta@igb.cnr.it (A.G.); c.scocco@studenti.unina.it (C.S.); 5 Department of Cell Biology, Physiology and Immunology, University of Córdoba, 14071 Córdoba, Spain; b32locaj@uco.es; 6 Robert and Monica Jacoby Center for Breast Health, Division of Hematology and Medical Oncology, Mayo Clinic, Jacksonville, FL 32224, USA; chumsri.saranya@mayo.edu; 7 Task Force for Microbiome Studies, University of Naples Federico II, 80138 Naples, Italy

**Keywords:** microbiota, microbiome, probiotics, *L. rhamnosus*, *L. paracasei*, metastasis, fermentation, breast tumor microenvironment

## Abstract

Breast cancer is the most common cancer in women, causing more than 600,000 deaths every year. The human microbiome is increasingly recognized as a key regulator of cancer initiation, progression, and therapeutic response. Postbiotics—defined as non-viable microbial cells and/or their structural components and metabolites that confer health benefits—are emerging as promising and safer alternatives to live probiotics in oncology. This review provides a comprehensive mechanistic overview of the potential role of postbiotics against cancer, with a specific focus on breast cancer. Preclinical evidence demonstrates that selected postbiotics exert dose- and time-dependent anticancer effects against multiple breast cancer subtypes by modulating key oncogenic pathways (such as PI3K/AKT and NF-κB) and inducing epigenetic regulation through histone deacetylase inhibition. Beyond direct effects on tumor cell proliferation and apoptosis, postbiotics influence the breast cancer microenvironment by reshaping cytokine networks, suppressing pro-metastatic inflammation, and enhancing antitumor immune responses through the activation of NK cells and T cells. We also provide emerging links between microbiome composition, estrobolome activity, and breast cancer subtype-specific biology. Despite these encouraging findings, the clinical translation of postbiotics in oncology remains limited. Currently, only one registered clinical trial investigates postbiotics in the oncology setting (melanoma), and no clinical trials have specifically evaluated postbiotics in breast cancer patients. This highlights a substantial translational gap between preclinical evidence and clinical application. Accordingly, well-designed, tumor-specific clinical trials are urgently needed to validate the safety, efficacy, and therapeutic potential of postbiotics as novel strategies in personalized breast cancer management.

## Simple Summary

Breast cancer is a major global health challenge. Recent research highlights the human microbiome’s crucial role in cancer development and treatment response. Postbiotics—beneficial compounds derived from non-viable microbes—have emerged as safer alternatives to live probiotics in oncology. This review explores the mechanistic impact of postbiotics in breast cancer, detailing how they inhibit tumor growth, regulate oncogenic pathways, and reshape the immune microenvironment. It also discusses the links between the microbiome, estrobolome activity, and specific tumor subtypes, showcasing how these promising preclinical findings are translating into ongoing clinical trials.

## 1. Introduction

Breast cancer (BC) is one of the most common cancers in women worldwide [1]. Human microbiota, particularly the gut microbiota, is a complex and dynamic community of microorganisms that play a crucial role in maintaining host homeostasis, which is fundamental for maintaining body health [2]. It influences a wide range of physiological functions, including immune modulation, metabolic regulation, and protection against pathogens and environmental factors by modulating the integrity of the gut barrier [3,4]. Alterations in the microbial composition of the gut, oral cavity, and breast tissue—collectively known as dysbiosis—have been increasingly associated with various diseases, including BC [5]. Beyond these specific niches, emerging evidence highlights the critical relevance of the oral–gut microbiome axis in BC pathogenesis. Oral dysbiosis and the subsequent translocation of oral pathogens or their metabolites can influence systemic inflammation and tumor development, suggesting that the oral microbiome is an integral part of the systemic environment favoring BC progression [6,7].

Since the early conceptualization of the microbiome–cancer axis proposed by Plottel and Blaser [8], accumulating evidence has indicated that microbiota composition changes during tumor development, and in some instances, these alterations may even precede tumor formation. These findings highlight that modulation of microbiota may represent not only a risk indicator, but also a potential therapeutic target. In this context, strategies aimed at restoring microbial balance, including the use of probiotics and postbiotics, are gaining increasing interest for their potential applications in cancer prevention and adjuvant therapy [9].

**Figure 1:**
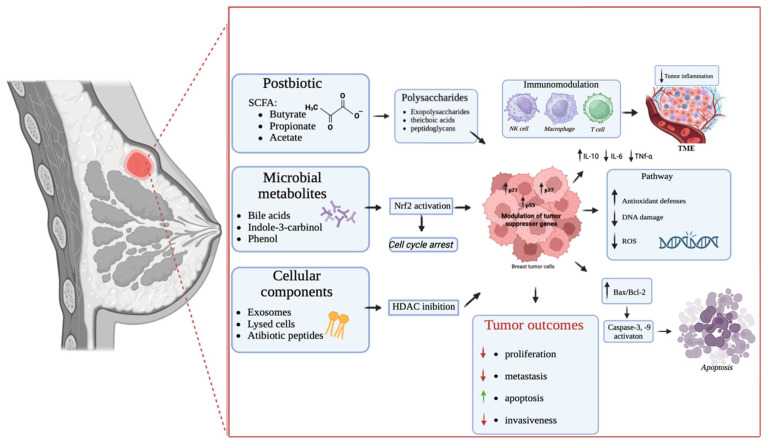
Postbiotics elicit different mechanisms for limiting BC progression. Postbiotics, microbial metabolites, and cellular components modulate multiple molecular pathways in BC cells and within the tumor microenvironment. These bioactive compounds promote immunomodulation (via NK cell, macrophage, and T cell activation), leading to reduced tumor-associated inflammation; intracellular signaling (via Nrf2 activation, which boosts antioxidant defenses and reduces reactive oxygen species and DNA damage); epigenetic regulation (histone deacetylase inhibition leads to upregulation of tumor suppressor genes, such as p21, p27, and p53); and induction of apoptosis (increasing levels of Bax/Bcl-2 ratio combined with the activation of Caspase-3 and Caspase-9). Collectively, these mechanisms contribute to reduced tumor proliferation, metastasis, and invasiveness and increased apoptotic cell death.

Probiotics, defined as live microorganisms that confer health benefits when administered in adequate amounts, have been extensively studied for their ability to restore microbial balance and modulate host responses [10]. More recently, attention has shifted to postbiotics, defined as preparations of inanimate microorganisms and/or their components and metabolites that confer health benefits to the host [11,12]. These include short-chain fatty acids (SCFAs), bacterial peptides, cell wall fragments, exopolysaccharides (EPS), and enzymes, many of which display anti-inflammatory, antioxidant, or immunomodulatory activities [11,13] (Figure 1). This distinction is crucial in oncology, as postbiotics offer a superior safety profile, stability, and standardization, avoiding the risks associated with administering live microbes to immunocompromised patients.

Postbiotics may reproduce some of the beneficial effects of probiotics while offering several practical advantages, including improved safety profile, stability, and standardization, especially important in immunocompromised or critically ill individuals, such as cancer patients [14,15]. Unlike probiotics, postbiotics do not require microbial colonization or survival in the gastrointestinal tract to exert their effects, thereby reducing potential risks linked to administering live microorganisms [16]. Increasing evidence highlights the pivotal role of the gut microbiota in cancer development and progression, influencing processes such as immune modulation, hormone metabolism, and inflammation [17,18]. In BC specifically, microbiota-derived metabolites have been shown to affect tumor behavior, therapeutic responsiveness, and systemic immune regulation [17]. These findings support the potential of postbiotics to serve as future precision tools in microbiota-informed oncology [19].

This narrative review focuses on the emerging evidence on the role of postbiotics in cancer, with a particular focus on BC. We analyzed the types and sources of postbiotics, summarized in vitro findings on BC cell lines, and discussed the molecular mechanisms involved in their anticancer effects. By examining these dimensions, we aimed to illuminate novel, targeted strategies for adjunctive treatment—an emerging frontier in microbiome-based cancer therapy.

To provide a comprehensive and transparent overview of the available evidence, a literature search was conducted using PubMed, Scopus, and Web of Science databases up to the time of manuscript preparation. The search strategy included combinations of the following keywords: “postbiotics”, “microbiome”, “breast cancer”, “cancer”, and “microbial metabolites”. Articles were selected based on their relevance to the topic, with priority given to recent studies and those providing mechanistic, preclinical, or clinical insights. Reference lists of selected articles were also screened to identify additional relevant studies. Non-English publications, conference abstracts without full text available, and studies not directly related to postbiotics in cancer were excluded. This review was conducted as a narrative synthesis and does not follow a formal systematic review protocol; however, this approach was designed to enhance transparency and reproducibility.

## 2. Overview of Postbiotic Categories

Postbiotics can be classified into five main categories according to their nature and composition, each potentially exerting distinct anticancer effects:

- Soluble metabolites (including SCFAs such as butyrate, propionate, and acetate; conjugated fatty acids; secondary bile acids (BAs); indoles derived from tryptophan metabolism; and polyamines) have been widely associated with modulation of inflammation and apoptosis in tumor cells [18]. Butyrate acts as a histone deacetylase (HDAC) inhibitor, leading to cell cycle arrest and programmed cell death in colorectal cancer cells [16,17]. Moreover, elevated fecal or systemic SCFA concentrations have been associated with improved response to immune checkpoint inhibitors in patients with solid tumors [18].
- Structural macromolecules, including peptidoglycan, lipoproteins, lipoteichoic acids (LTAs), bacterial DNA rich in CpG motifs, and bacteriocins or antimicrobial peptides, interact with immune receptors and contribute to antitumor immune responses [12]. Some bacteriocins also show direct cytotoxic activity against cancer cell lines [20].
- Biomolecules, such as EPS, bioactive proteins, enzymes, and peptides, may contribute to tumor suppression through antioxidants, immunomodulatory, and antiproliferative mechanisms [21,22]. EPS produced by Lactobacillus and Bifidobacterium species have been shown to suppress the PI3K/Akt pathway, increase the BAX/Bcl2 ratio, and induce apoptosis in colon and BC models [22].
- Nanoparticles, represented by bacterial extracellular vesicles, act as delivery systems for bioactive compounds (i.e., lipids, proteins, nucleic acids, and metabolites), influencing tumor microenvironment (TME) communication [20,23]. These vesicles emerge as key mediators in host–microbiota interactions and can modulate tumor growth by affecting immune responses and gene expression [23].
- Finally, inactivated cells, also called paraprobiotics—obtained through heat, irradiation, or pressure treatments—preserve immunomodulatory components capable of eliciting beneficial immune responses even in the absence of viable microorganisms [24]. Several in vitro and in vivo studies have demonstrated that inactivated bacterial cells can reduce tumor proliferation and stimulate antineoplastic immune responses [25,26].

Overall, each postbiotic category exhibits a specific mechanism of action: metabolites primarily affect tumor and epigenetic pathways, whereas macromolecules and biomolecules modulate immune and oxidative responses; nanoparticles and inactivated cells represent promising tools for novel anticancer strategies.

## 3. Importance of Postbiotics Dosage and Exposure Time

The anticancer potential of postbiotics has been widely explored in pre-clinical cancer models, particularly in breast and gastrointestinal cancers. The activity of postbiotics depends significantly on the type of bacterial strain used, the preparation method (e.g., heat-killed cells, lysates, or cell-free supernatants), the administered dose, and the duration of exposure (Table 1). These parameters determine cellular outcomes such as growth inhibition, apoptosis induction, cell cycle arrest, and immunomodulatory effects.

In BC models, *Lactiplantibacillus plantarum*, *L. rhamnosus*, *L. brevis*, and *L. casei* have been extensively studied. Notably, *L. plantarum* 5BL, a vaginal commensal bacterial strain, was found to induce apoptosis and thereby showed significant anticancer activity against some cancer cell lines, including MCF-7 [9]. Chuah et al. [27] demonstrated that cell-free supernatants of *L. plantarum* reduced MCF-7 cell viability at concentrations ranging from 5 to 30% (*v*/*v*) for 24–72 h of treatment. The maximal effects were observed after prolonged exposure (72 h), concomitant with increased apoptosis and oxidative stress markers. Comparable findings were reported for cell-free supernatants from *L. brevis* and *L. casei*, which, at concentrations of 1–2 mg/mL for 48–72 h, markedly decreased proliferation and induced apoptotic cell death in MCF-7 cells [28].

Heat-killed preparations have shown variable efficacy depending on dose and bacterial strain. Heat-killed *Enterococcus faecalis* and *Staphylococcus hominis* reduced MCF-7 cell proliferation at concentrations ≥ 0.025–0.2 mg/mL after 24–72 h, with more pronounced effects at higher doses and longer exposure times [29].

**Table 1:** Evidence on the effect of different postbiotic preparations on cancer cell lines.

Type of Postbiotic	Bacterial Source	Cell Line	Dose and Exposure Time	Observed Effect	Reference
Cell-free supernatant	*L. plantarum*	MCF-7	5–30% (*v*/*v*); 24–72 h	reduced cell viability	[27]
Heat-killed bacteria	*Enterococcus faecalis*	MCF-7	0.025–0.2 mg/mL; 24–72 h	antiproliferative effect	[29]
	*Staphylococcus hominis*				
Cell-free supernatant	*L. paracasei*	SKBR3	5–20 mg/mL; 24–48 h	dose-dependent upregulation of surface HLA class I expression	[30]
Probiotic lysates	*L. acidophilus*	MDA-MB-231	0.05–0.2 mg/mL; 24 h	antiproliferative effect	[31]
	*L. casei*				
	*Bifidobacterium lactis*				
	*L. rhamnosus*				
Heat-killed bacteria	*L. brevis*	gastric cancer AGS cells	9 log CFU/mL; 44 h	apoptotic body formation	[32]
Bioactive peptidoglycan	*L. paracasei*	gastric cancer AGS cells	1–10% (*v*/*v*)	inhibition of cell viability	[33]

In human epidermal growth factor receptor 2-positive (HER2+) SKBR3 cells, postbiotics appear to exert not only cytostatic but also immunomodulatory effects. Cell-free supernatants from *L. paracasei* administered at 5–20 mg/mL for 24–48 h induced a dose-dependent upregulation of surface HLA (Human Leukocyte Antigen) class I expression, suggesting enhanced immunogenicity and potential for immune-mediated tumor suppression [30]. Triple-negative BC (TNBC) models, such as MDA-MB-231, are particularly sensitive to postbiotic lysates. Lysates derived from *L. acidophilus*, *L. casei*, *Bifidobacterium lactis*, and *L. rhamnosus* in the range of 0.05–0.2 mg/mL significantly suppressed proliferation within 24 h across all tested doses [31,34].

In colorectal cancer cell lines, cell-free supernatants derived from *L. casei*, *L. brevis*, and *L. paracasei* have demonstrated dose-dependent inhibition of cell growth following 24–72 h of treatment. Depending on the study, postbiotics were administered either as increasing proportions of cell-free supernatants (approximately 20–70% *v*/*v*) or using strain-specific preparations, resulting in G0/G1 cell cycle arrest, increased intracellular reactive oxygen species (ROS) production, and apoptosis [28].

Similarly, in gastric cancer Adenocarcinoma Gastric Stomach (AGS) cells, heat-killed *L. brevis* at a bacterial load of 9 log CFU/mL significantly reduced proliferation after 44 h, accompanied by chromatin condensation and apoptotic body formation [32]. Additionally, purified postbiotic components also exhibit pronounced anticancer effects. For instance, bioactive peptidoglycan isolated from *L. paracasei* at 1–10% (*v*/*v*) suppressed AGS cell viability and inhibited COX-2 expression via NF-κB signaling modulation [33].

Collectively, these studies indicate that postbiotics can exert dose- and time-dependent anticancer effects across different cancer models. These findings support their potential integration into multimodal anticancer strategies, either as adjuvants to conventional therapies or as modulators of tumor immunogenicity. However, the marked heterogeneity in dosing regimens highlights the need for standardized reporting and optimization frameworks to improve comparability and translational applicability.

The focus on non-viable preparations is essential; the heterogeneity in reported outcomes emphasizes the need to standardize inactivation methods to ensure that structural components (like peptidoglycans) effectively interact with host receptors without the risks associated with live microbes.

### Methodological Challenges in Breast Microbiome Research

While the association between the microbiome and BC is increasingly recognized, several methodological challenges must be considered when interpreting current evidence. Unlike the gut, breast tissue represents a low-biomass microbial niche, making it inherently difficult to distinguish resident microorganisms from contaminants [35]. Consequently, sequencing-based studies are particularly susceptible to environmental and reagent-derived DNA contamination (“kit-ome”), which may substantially bias microbial profiling if appropriate controls are not implemented [35,36]. In addition, tissue sampling poses significant technical challenges, as breast biopsies and surgical specimens are highly vulnerable to contamination from the skin microbiota during collection and processing [37,38]. Therefore, standardized sampling protocols, rigorous negative controls, and harmonized analytical pipelines are essential to ensure reliable characterization of the breast microbiome and to improve reproducibility in the future [35,39].

## 4. Mechanistic Pathways Linking the Microbiome to Breast Cancer Progression

A growing body of evidence suggests that the microbiome influences multiple hallmarks of BC, acting through metabolic, immunological, and molecular pathways. The most consistently described mechanisms involve modulation of tumor cell growth and survival, cytokine signaling related to metastasis, inflammatory responses, and remodeling of the TME.

### 4.1. Tumor Cell Growth and Survival

Microbial communities and their metabolites exert direct and indirect effects on BC cell proliferation, apoptosis, and metabolic reprogramming. Dysbiosis has been associated with enhanced activation of oncogenic pathways such as PI3K/AKT, NF-κB, and IL6/JAK/STAT3, promoting tumor growth and resistance to cell death [40,41]. In addition, dysbiosis may trigger a DNA damage-mediated oncogenic axis exemplified by the IL-6/TNF-α–SMOX–DDR pathway. Recent studies have shown that specific gut pathogens, including enterotoxigenic *Bacteroides fragilis* (ETBF), *Fusobacterium nucleatum*, and *Escherichia coli* pks+, increase the expression of spermine oxidase (SMOX), a key enzyme involved in polyamine metabolism, whereas nonpathogenic bacteria do not exert this effect. Increased SMOX activity promotes ROS production, γ-H2AX foci formation, and disruption of the DNA damage response (DDR). Furthermore, elevated IL-6 and TNF-α levels further amplify SMOX activation, creating a pro-tumorigenic inflammatory loop. Pharmacological inhibition of SMOX with MDL72527 or SXG-1 blocks these effects, reducing ETBF-induced tumorigenesis and identifying the IL-6/TNF-α–SMOX–DDR axis not only as a key mediator of the oncogenic effects of pathogenic bacteria but also as a potential therapeutic target [42]. However, it is important to exercise caution in translating these findings; although pharmacological inhibition of SMOX has proven effective in blocking pathogen-induced tumorigenesis in experimental models, direct clinical data confirming the activation and functional role of this specific axis in human breast tissue are currently lacking. Further research is required to validate whether these preclinical observations hold true in the complex environment of human malignancy.

Conversely, specific commensal bacteria (e.g., *Lactobacillus, Bifidobacterium*, and butyrate-producing taxa) can suppress these pathways and influence hormone receptor signaling, contributing to reduce proliferative activity [43]. Recent studies suggest that certain postbiotic compounds produced by these commensal bacteria may modulate the expression of genes involved in the regulation of cell growth. SCFAs, particularly butyrate, act as HDAC inhibitors, inducing epigenetic reprogramming of cancer cells and upregulating tumor suppressor genes, such as p21 and p27, thereby promoting, apoptosis and genomic stability [44,45] (Figure 1). These effects suggest a potential role of postbiotics in modulating the mechanisms that control cell proliferation [45], acting directly on target sites without compromising the neighboring cells [15]. Microbial metabolites—including SCFAs (acetate, propionate and butyrate), indole derivatives, and secondary BAs—have emerged as key regulators of tumor biology. These compounds can alter epigenetic states, induce apoptosis, limit DNA damage, and enhance antitumor immune responses [46]. Collectively, these findings support the concept that microbiome-derived biochemical signals can significantly influence the survival dynamics of BC cells.

### 4.2. Modulation of Cytokines and Metastasis Prevention

The microbiome plays an essential role in shaping cytokine networks, cell signaling and immune surveillance, thereby influencing BC initiation, progression and metastatic potential. Key mechanisms include the sustained activation of pro-inflammatory cascades (STAT3/NF-κB) and the recruitment of immunosuppressive cells (M2 macrophages and MDSCs), collectively fostering a pro-tumorigenic and immune-evasive environment. Dysbiosis states often correlate with elevated levels of IL-6, IL-1β, TNF-α, and other pro-inflammatory mediators that facilitate epithelial-to-mesenchymal transition (EMT), angiogenesis, and dissemination [47] (Figure 1). Several probiotic and postbiotic interventions have been shown to downregulate IL-22 or modulate IL-17 and IL-23 signaling, thereby reducing tumor invasiveness and suppressing pro-metastatic immune profiles. In preclinical models, strains such as *L. plantarum*, *L. casei*, and *L. acidophilus* suppress the IL-23/Th17 axis, lowering IL-17 and IL-23 expression and rebalancing Th17/Treg responses—immune circuits well-known to promote tumor growth, angiogenesis, and metastatic dissemination [48].

Postbiotics may further reduce metastatic potential by decreasing matrix metalloproteinase (MMP) activity and inhibiting angiogenesis through VEGF downregulation mediated by neuropilin-1 suppression. In parallel, SCFA-mediated HDAC inhibition restores tumor suppressor activity, whereas AMPK modulation contributes to maintaining intestinal barrier integrity [49]. These mechanisms support the potential use of postbiotics as adjuvant therapies, capable of enhancing anticancer activity while minimizing off-target effects on healthy tissues. Postbiotic metabolites can also attenuate IL-17-producing cell populations and blunt inflammation-driven tumor-promoting circuits.

Although some engineered probiotics have been designed to deliver IL-22 for mucosal repair, these represent context-specific systems and highlight that microbial interventions can either enhance or suppress IL-22 depending on therapeutic intent [50]. Overall, accumulating evidence indicates that probiotic- and postbiotic-mediated modulation of IL-22, IL-17, and IL-23 can shift the TME away from inflammation-driven metastatic programs toward more effective antitumor immune responses. Through these immunomodulatory actions, regulation of cytokine production represents a promising microbiome-targeted strategy to inhibit metastasis in BC.

Nevertheless, several underlying mechanisms were first identified using live probiotic strains (e.g., *L. plantarum* and *L. casei*), and emerging evidence indicates that their postbiotic derivatives—such as cell-free supernatants or lysates—can elicit similar responses, acting as the actual precision tools in microbiota-informed oncology.

### 4.3. Inflammation

Chronic inflammation is a well-established driver of BC progression, and the microbiome represents a central regulator of inflammatory tone. Microbial-derived molecules can influence tumor-associated inflammation through the activation of pattern recognition receptors, particularly Toll-like receptors (TLRs), leading to downstream signaling pathways such as NF-κB activation and the production of inflammatory mediators, such as COX-2, prostaglandins, and ROS [51]. These inflammatory processes contribute to tumor initiation, proliferation, metastatic progression and therapy resistance. The inflammatory process typically occurs when pathogen-associated molecular patterns (PAMPs) are detected by pattern recognition receptors (PRRs) on antigen-presenting cells. Among these receptors, TLRs play a crucial role in sensing microbial components and regulating innate immune responses [52]. The TLR family comprises 13 members, each characterized by specific ligand recognition. For example, TLR1, TLR2, and TLR6 detect lipoproteins derived from Gram-positive bacteria, TLR4 recognizes lipopolysaccharide (LPS), and TLR5 uniquely responds to flagellin. TLR2 typically functions as a heterodimer with TLR1 or TLR6 [48]. The interaction between TLR2 and its ligand can activate a MyD88-dependent signaling pathway, culminating in NF-κB production, as demonstrated in studies conducted on dendritic cells [51]. Similarly, TLR9 recognizes unmethylated CpG motifs present in microbial DNA and activates MyD88-dependent pathways involving IRAK and TRAF6, ultimately promoting IκB degradation and NF-κB nuclear translocation [53,54,55]. Several studies have demonstrated different patterns of TLR expression in BC tissues and cell lines. One example is the differential expression of TLR2 and TLR3 among BC cell lines: TLR2 levels are higher in MDA-MB-231 cells compared to MCF-7 cells, whereas TLR3 expression is lower in the MDA-MB-231 cell line [56]. Growing evidence suggests that TLRs are involved in BC development and prognosis, with increased expression of TLR3, TLR4, and TLR9 being associated with reduced survival in BC patients [57]. Under conditions of microbial dysbiosis, TLR signaling in BC cells is upregulated, leading to activation of the NF-κB pathway and subsequent secretion of inflammatory cytokines that may promote tumor cell proliferation. Emerging evidence, however, indicates that NF-κB, although typically pro-inflammatory and associated with cell survival, can mediate apoptosis in a context-dependent manner. For instance, NF-κB is required for p53-dependent apoptosis [58]; overexpression of the NF-κB subunit p65/RELA in MCF-7 cells induces substantial cell death, suggesting a direct pro-apoptotic role [59]. Furthermore, TRAIL-mediated apoptosis in MCF-7 and MDA-MB-231 cell lines involves NF-κB activation, indicating that it may act as a co-regulator in apoptotic pathways [60]. NF-κB signaling is driven by increased expression of chemokines such as CXCL1, CXCL2, CXCL3, CXCL8, and CXCL10, which in turn enhances the release of inflammatory factors including IL-3, IL-4, IL-10, and IL-17, collectively contributing to BC progression [61].

Importantly, postbiotics may regulate these inflammatory pathways. Postbiotics include a wide range of bioactive compounds such as microbial metabolites, bacterial structural components, extracellular vesicles, enzymes, and other molecular mediators capable of interacting directly with host cells [62,63], and these compounds may influence PRR-mediated signaling, including TLR pathways, thereby modulating inflammatory responses and immune functions [16]. Among postbiotic metabolites, SCFAs represent the most extensively studied class involved in inflammation and cancer biology. Butyrate, acetate, and propionate regulate inflammatory responses through multiple mechanisms, including activation of free fatty acid (FFA) receptors (FFA2 and FFA3) and inhibition of HDACs. In addition, SCFAs modulate immune responses by regulating regulatory T cells (Tregs), dendritic cells, macrophage activity, and pro- and anti-inflammatory cytokine production, while promoting plasma B-cell proliferation and thereby contributing to a more protective immunological landscape [64,65]. SCFAs can reduce excessive NF-κB activation and pro-inflammatory mediator release, contributing to a less permissive TME [44,65]. In BC models, these metabolites have shown potential antitumor effects through apoptosis induction, oxidative stress modulation, and regulation of estrogen receptor-related pathways [66,67]. Other microbial-derived postbiotic components associated with postbiotic preparations, including extracellular vesicles and microbial nucleic acids, may also modulate tumor-associated inflammation by interacting with host immune receptors [23]. For example, bacterial DNA containing unmethylated CpG motifs can activate TLR9 signaling independently of bacterial viability [55]. Collectively, these findings support postbiotics as key molecular mediators between the microbiome and the TME. Nevertheless, despite promising preclinical evidence of anticancer and immunomodulatory effects in BC models [28,45], their clinical translation remains limited. Further studies are required to define their bioavailability, formulation, dosing, and patient-specific determinants of efficacy.

### 4.4. Breast TME

The TME is a dynamic and multifaceted ecosystem comprising heterogeneous cell populations, extracellular matrix (ECM) components, and soluble factors that interact bidirectionally with malignant cells. Its cellular constituents—including cancer-associated fibroblasts (CAFs), tumor-associated macrophages (TAMs), regulatory lymphocytes, endothelial cells, neutrophils, macrophages, natural killer cells, dendritic cells, and myeloid-derived suppressor cells—communicate with tumor cells through paracrine and juxtacrine signaling. These interactions regulate proliferation, angiogenesis, invasion, immune modulation and therapeutic resistance. Non-cellular components such as cytokines, chemokines, growth factors, and ECM proteins also shape tissue stiffness, immune cell infiltration, and cellular phenotypes within the TME [68].

A defining feature of the TME is its metabolic complexity. CAFs and cancer-associated adipocytes (CAAs) support tumor growth by supplying metabolites and modulating nutrient availability, while hypoxic niches activate HIF 1α, promoting angiogenesis and fostering immunosuppressive phenotypes. These metabolic-immune interactions skew immune responses toward tumor-promoting states and contribute to therapeutic resistance, underscoring the need to understand how metabolic cues shape the TME [68].

Growing evidence indicates that the microbiome significantly influences these processes. Microbiota-derived metabolites regulate the activity of immune cells, fibroblasts, endothelial cells, and ECM components, thereby affecting inflammation, proliferation, angiogenesis, EMT, invasion, and immune evasion [69]. Dysbiosis is associated with increased infiltration of immunosuppressive cell types such as Tregs and M2 macrophages, reduced cytotoxic T-cell activity, and enhanced angiogenesis. TAMs frequently display an M2-like phenotype, secreting VEGF, IL-10, and TGF β, which promote angiogenesis and tumor survival, whereas M1 macrophages and activated CD8^+^ T cells contribute to antitumor immunity when appropriately activated. Regulatory T- and B-cells further suppress cytotoxic antitumor responses. Emerging immunotherapeutic strategies—including checkpoint inhibitors and adoptive cell therapies—seek to remodel this immune landscape to enhance antitumor responses, particularly in aggressive subtypes such as TNBC [70].

Postbiotics and microbial-derived metabolites further modulate TME by influencing stromal remodeling, vascular integrity, and metabolic crosstalk between tumor and stromal cells. In this context, SCFAs act as tumor suppressors across a wide range of cancer types [71]. They activate GPR41 and GPR43 in intestinal epithelial cells, triggering mitogen-activated protein kinase (MAPK) signaling and promoting controlled chemokine and cytokine production [72]. SCFAs have also been shown to preserve the mucosal barrier while reducing levels of immunomodulators, such as COX-2-derived prostaglandins, which are linked to inflammation and tumor-associated growth in human MCF7 BC cells [71].

Within the TME, soluble mediators with anti-inflammatory or pro-apoptotic functions, such as TRAIL, IL-10, and TGF-β, can hinder carcinogenesis. Furthermore, postbiotics stimulate IL-12 production in macrophages and support mucosal and systemic immune homeostasis, contributing to the suppression of inflammation-driven carcinogenesis [62]. Collectively, these interactions highlight the capacity of the microbiome and its postbiotic metabolites to shape the TME toward a less inflammatory and more immunocompetent state, thereby limiting carcinogenesis and supporting antitumor immune responses.

## 5. Variations in the Microbiome in Relation to Breast Cancer Type

Emerging evidence highlights that the gut microbiome plays a pivotal role in the pathophysiology of BC, influencing both hormonal regulation and systemic inflammation [73]. Several studies have reported that women diagnosed with BC frequently exhibit reduced gut microbial diversity compared to healthy controls [74].

A landmark case–control study by Yaghjyan et al. [74] further demonstrated that postmenopausal women with newly diagnosed, untreated BC had significantly lower fecal microbial alpha diversity and a distinct overall microbial composition (beta diversity) than matched healthy controls. Importantly, in healthy women, total urinary estrogens were positively correlated with microbial diversity, whereas this association was absent in BC patients. These findings suggest that the physiological relationship between gut microbiota and systemic estrogen metabolism may be disrupted in the presence of malignancy. Yaghjyan et al. [74] emphasized that BC associated with dysbiosis appears to involve community-wide alterations in microbial structure, supporting the concept that functional capacity, rather than individual taxa alone, may be central to disease risk. Bacterial taxa involved in estrogen metabolism, such as *Clostridium* spp. and *Bacteroides* spp., are often enriched in BC patients, reflecting an increased capacity for β-glucuronidase-mediated deconjugation and reactivation of estrogens in the gastrointestinal tract [75]. Through this mechanism, conjugated estrogens excreted via bile may be reactivated and reabsorbed into circulation, increasing systemic estrogen exposure. However, the mechanistic link between microbial β-glucuronidase activity and systemic estrogen levels warrants a more critical evaluation. While the biochemical pathway of estrogen deconjugation and reabsorption is well-characterized, its quantitative contribution to the total systemic estrogen pool—especially when compared to endogenous production in pre- and post-menopausal contexts—remains to be fully elucidated. Notably, clinical observations show that the positive correlation between microbial diversity and urinary estrogens seen in healthy women is disrupted or entirely absent in BC patients. This suggests that malignancy-associated dysbiosis does not simply “increase” estrogen levels in a linear fashion but rather represents a profound functional breakdown of the microbiota–estrogen axis.

Furthermore, this gut-level disruption may be further exacerbated by the oral–gut microbiome axis, through which oral pathogens can contribute to a pro-tumorigenic inflammatory tone [10]. Finally, since most evidence is derived from observational case–control studies, establishing a definitive causal relationship remains a significant challenge for the field.

This concept has given rise to the term “estrobolome”, which refers to the collection of microbial genes capable of metabolizing estrogens [8]. The functional activity of the estrobolome may modulate circulating estrogen levels and thus influence hormone-driven carcinogenesis. An estrobolome enriched in β-glucuronidase and sulfatase activity may increase levels of biologically active estrogens, thereby promoting prolonged stimulation of estrogen receptors and potentially contribute to the development of estrogen receptor-positive (ER+) breast tumors [66]. This mechanism may be particularly relevant in postmenopausal women, for whom peripheral and microbial estrogen metabolism contributes substantially to systemic hormone levels.

Beyond endocrine effects, gut microbial dysbiosis may also promote carcinogenesis through inflammatory, genotoxic, and immune-modulatory pathways. Increased relative abundances of *Escherichia coli*, *Enterococcus faecalis*, and *Ruminococcus* spp. have been reported in BC patients [70]. Certain strains of *E. coli*, for example, can produce genotoxic metabolites that induce DNA damage, while other species may contribute to chronic low-grade inflammation or altered immune surveillance. Conversely, reductions in beneficial genera such as *Faecalibacterium* and *Lactobacillus* have been associated with decreased production of SCFAs, impaired epithelial barrier integrity, and diminished anti-inflammatory signaling.

Taken together, these findings support a model in which gut microbiome alterations in BC are characterized not only by reduced diversity but also by functional shifts affecting estrogen metabolism, immune regulation, and inflammatory tone. However, although these associations suggest a potential contribution of microbiome alterations to BC development and progression, current evidence does not establish direct causality. Microbial changes may represent both potential contributors to tumor biology and consequences of disease-related alterations in the host environment. The disruption of the microbiota–estrogen axis observed in BC patients suggests that microbial community structure and estrobolome activity may represent important contributors to hormone-dependent tumorigenesis [76].

Distinct microbial signatures have been reported among different BC subtypes, underscoring the complexity of microbe–tumor interactions:

- ER+ tumors have been reported in some studies to be associated with enrichment of *Clostridium* and *Bacteroides* species that participate in estrogen recycling via β-glucuronidase activity, supporting estrogen-driven carcinogenesis [75]. Some studies also link *Ruminococcus* and *Eubacterium* to increased estrogen metabolism and chronic inflammation in ER+ disease [77].
- ER− (Estrogen Receptor-Negative) and TNBC subtypes exhibit a different microbial landscape, often characterized by increased abundance of pro-inflammatory genera such as *Escherichia, Pseudomonas*, and *Fusobacterium*, which can contribute to immune evasion and tumor aggressiveness. TNBC tissues have been reported to harbor *Methylobacterium radiotolerans* and *Sphingomonas yanoikuyae*, potentially influencing redox signaling and response to therapy [78].
- HER2+ tumors show distinct microbial associations as well, including enrichment of Actinobacteria and *Firmicutes*, possibly reflecting interactions between microbial metabolites and HER2 signaling pathways [79].

A comparative summary of these subtype-specific microbiome features is provided in Table 2. Collectively, these subtype-specific associations raise the hypothesis that the microbiome may contribute to shaping tumor phenotype and treatment responsiveness; however, most underlying evidence is correlative, derived from cross-sectional studies, and does not establish causality. Differences in microbial metabolic capacity—such as SCFA production, BA transformation, and estrogen reactivation—may partly contribute to the biological heterogeneity observed among BC subtypes, though this remains to be experimentally validated.

Taken together, current evidence supports a model in which the gut microbiome–estrogen axis, mediated by the metabolic capacity of the estrobolome, plays a pivotal role in BC susceptibility and progression. Understanding these microbiota–host interactions may open new avenues for preventive and therapeutic strategies, including dietary modulation, probiotic or postbiotic supplementation, and microbiome-targeted interventions.

## 6. Mechanisms of Postbiotics Against Breast Cancer

Several mechanisms have been proposed to explain the effects of postbiotics in the human body, including: (i) modulation of the resident microbiota; (ii) enhancement of epithelial barrier functions; (iii) modulation of local and systemic immune responses; (iv) modulation of systemic metabolic responses; and (v) systemic signaling via the nervous system [12]. However, the molecular mechanisms underlying their anticancer activity differ according to the specific class of postbiotic and its biological properties.

### 6.1. Soluble Metabolites

In the postbiotic context, soluble metabolites are low-molecular-weight compounds secreted or released by gut or food-grade microorganisms during their growth and fermentation in microbiological cultures [80]. They can cross biological barriers, enter systemic circulation, and influence distant organs including the mammary gland [63,81].

### 6.2. Short-Chain Fatty Acids

SCFAs, principally acetate, propionate and butyrate, are generated through the bacterial fermentation of dietary fibers and resistant starches by anaerobic bacteria such as *Bacteroides, Clostridium,* and *Faecalibacterium prausnitzii* [82]. Once produced, a portion of SCFAs is absorbed into the portal circulation and, after hepatic passage, can reach the systemic bloodstream and thereby distant organs. Although direct quantification of mammary-tissue penetration is limited, the presence of SCFA-responsive receptors (such as GPR41, GPR43, and GPR109A) in mammary cells supports the concept that SCFAs can act on the mammary gland [83]. The complex pharmacology of these FFA receptors, particularly GPR41 (FFA3) and GPR43 (FFA2), is fundamental for understanding how microbial metabolites trigger systemic signaling [64].

Several studies have demonstrated concentration-dependent antitumor effects of SCFAs in BC cells, including a progressive reduction in cell viability, which appears to occur independently of GPR43 or GPR41 receptor activation [65]. Different mechanisms have been reported to induce this effect by its HDAC inhibitor action, such as increased cell cycle arrest, increased ROS levels and reduced mitochondrial membrane potential, and increase in intracellular Ca^2+^ or phosphorylation of MAPK p38 [84]. At the molecular level, SCFAs inhibit key oncogenic pathways, including the PI3K/AKT/mTOR and NF-κB signaling cascades, thereby reducing tumor growth and survival [67] and disrupting the Warburg effect, characterized by enhanced aerobic glycolysis in cancer cells [85].

### 6.3. Conjugated Fatty Acids

Among lipid-derived postbiotics, conjugated fatty acids (including mainly conjugated linoleic and, to a lesser extent, conjugated linolenic and docosahexaenoic acids) have demonstrated antiproliferative activity and reduced tumor growth effects in BC models and, in some models, limited metastatic dissemination [86,87]. Several bacterial phyla have been identified as producers of conjugated fatty acids, such as actinobacteria (genus *Bifidobacterium*) [88] and *Firmicutes* (genus *Lactobacillus*), or even proteobacteria [89]. Nevertheless, direct evidence demonstrating that conjugated fatty acids produced by the human microbiota influence BC is lacking. The molecular pathways have been described to act through modulation of the Peroxisome Proliferator-Activated Receptor (PPAR) pathway, oxidative stress pathway, and lipid metabolism genes [90].

### 6.4. Secondary Bile Acids

BAs belong to cholesterol-derived sterols from hepatic metabolism. In the gut, these BAs are used by the microbiome producing dehydroxylated primary BAs or secondary BAs, mostly in the large bowel [91]. The main secondary BAs in humans are lithocholic acid (LCA), deoxycholic acid (DCA) and, to a lesser extent, ursodeoxycholic acid (UDCA) [91]. In the context of BC, secondary BAs have been reported in low levels due to a reduction in hepatic BA production [92]. Moreover, DCA is accumulated in human breast tumors, specifying that the tumor-suppressive association (reduced proliferation and improved survival) derives from clinical/tissue-level correlative data in bulk tumor samples [62], whereas the tumor-promoting effects (AKT/cyclin-D1 activation and FXR-mediated ceramide downregulation) were observed in specific experimental settings—namely physiological DCA concentrations in bulk tumor cell populations versus CD44+ stem-like subpopulations, respectively [93].

### 6.5. Indole-Derived Tryptophan Metabolites

The essential amino acid tryptophan can be metabolized by the gut microbiota through the indole pathway, generating metabolites such as Indole-3-Lactic Acid (ILA), Indole-3-Propionic Acid (IPA), Indole Acetic Acid (IAA), Indole-3-Aldehyde (IAld) and indole-3-pyruvic acid (IPyA), which have emerged as important regulators of tumor progression [94]. In BC, IPyA can directly inhibit the transcription of UHRF1, thereby suppressing the growth of breast and cervical cancer cells in an AMPK signaling pathway-mediated manner, and it has been reduced in BC patients by *Prevotella copri* [95]. Moreover, BC cell lines, including T47D, have been shown to be sensitive to IPA and IAA [96]. In this sense, it has been reported that IPA has a pivotal role in regulating the progression but not the initiation of the disease [97] and in potentiating immunotherapy [98].

### 6.6. Polyamines

Polyamines are the product of catabolism of amino acids by cells or microbiota. In fact, microbiota-derived polyamines can cross the intestinal epithelium and enter the systemic circulation, acting as metabolic signals that modulate cellular and immune functions, influence tissue homeostasis, and potentially alter processes relevant to carcinogenesis [99,100]. Major polyamines include putrescine, cadaverine, spermidine and spermine [101]. In BC cells, some of these polyamines have demonstrated the pro-tumoral effect, with cancer cells being their productors [102]. However, polyamine produced by microbiome, such as cadaverine, reduces EMT, invasion, and metastasis in BC models and acts through Trace Amine-Associated Receptors (TAARs) [103].

### 6.7. Bacterial-Derived Sugar

Beyond commonly studied soluble metabolites, probiotic bacteria also produce various sugar-based compounds capable of influencing host physiology and disease outcomes. Among these, trehalose—a non-reducing disaccharide synthesized by Lactobacillus species—is emerging as a notable anticancer modulator. Recent studies have highlighted its multifaceted activity, showing that this molecule, which cannot be synthesized by mammalian cells, is directly involved in autophagy induction and modulation of tumor metabolism in ER+ BC cells in vitro and in murine tumor models in vivo. Nevertheless, the precise molecular mechanisms underlying these effects remain under investigation. Moreover, the potential of trehalose is further supported by Li et al.’s investigation in TNBC [103], where it significantly delays and reduces tumor growth both in vitro and in vivo.

### 6.8. Structural Macromolecules

Structural macromolecules and biomolecules produced by the gut microbiota, such as cell wall components, EPS, peptidoglycans, and surface-associated proteins, are also considered postbiotics, as they can exert biological effects, including anticancer effects, on host tissues even in the absence of viable bacteria [48].

### 6.9. Peptidoglycan

Peptidoglycans are structural components of the bacterial cell wall composed of glycan chains cross-linked by short peptides. These microbiome-derived peptidoglycans could have effects on cancer cells. In BC, peptidoglycans from bacterium *Staphylococcus aureus* have been found to reach breast tissue through extracellular vesicles or systemic circulation, activating TLR2 receptors and promoting inflammation, cell proliferation, and tumor progression in BC cells [104].

Furthermore, peptidoglycan fragments derived from *Lactobacillus* species such as *L. acidophilus, L. casei*, and *L. rhamnosus* have been shown to enhance cytokine release by engaging TLR4 signaling pathways, thereby amplifying LPS-dependent immune activation [15].

### 6.10. Lipoteichoic Acid

LTA is an essential structural component in the cell wall of Gram-positive bacteria which is recognized by TLR2, triggering immunomodulatory responses [105]. No direct evidence links LTA to BC progression; however, endocrine-targeting therapies, such as tamoxifen, have been shown to reshape the breast microbiome, increasing LTA-positive Gram-positive bacteria, which correlate with reduced tumor proliferation and may contribute to a lower risk of ER+ BC [43].

### 6.11. CpG Motifs of Bacterial DNA

Foundational research has established that unmethylated CpG motifs from bacterial DNA can activate TLR9-mediated inflammatory signaling pathways [54,106]. Because TLR9 is also expressed in BC cells [57], microbial CpG DNA may influence the TME [57]. However, direct evidence of bacterial CpG DNA driving BC progression in vivo remains limited. Similar to conjugated fatty acids, direct evidence linking LTA or bacterial CpG DNA motifs to BC progression in vivo remains limited. These molecular pathways, while mechanistically plausible in laboratory settings, represent another significant translational gap that needs to be addressed through clinical research.

### 6.12. Biomolecules

#### 6.12.1. Exopolysaccharides

EPS from bacteria of different origin, such as marine-derived bacteria, have demonstrated antitumor effects in BC in vitro and in vivo by mitochondrial apoptosis pathways (BAX/Bcl-2, caspase-3), cell cycle arrest, modulation of NF-κB/STAT1 signaling, and immunomodulatory/antioxidant effects [107,108] (Figure 1). Beyond their direct effects on tumor cells, EPS may influence the TME by regulating immune cell activity, oxidative stress responses, and inflammatory signaling, suggesting that their biological effects extend beyond simple cytotoxicity [108]. However, EPS activity is highly dependent on molecular structure, bacterial origin, and experimental context, which may contribute to variability among studies [21,22]. On the other hand, the commensal bacterium *Bacillus subtilis* on BC has been reported as a potential probiotic, and its EPS treatment (short-term) can inhibit proliferation in certain BC cells. Conversely, prolonged exposure may promote resistance, enhance aggressive phenotypes, and stimulate pro-inflammatory pathways, highlighting the context-dependent effects of EPS [108].

#### 6.12.2. Bacteriocins

Similarly, bacteriocins represent another important class of proteinaceous postbiotics with emerging anticancer potential. These antimicrobial peptides are synthesized by bacteria and can preferentially interact with cancer cell membranes. Based on their bacterial origin and structural characteristics, bacteriocins can be classified into different groups, including microcins, colicins, and tailocins from Gram-negative bacteria, and classes I–IV from Gram-positive bacteria [109]. Several bacteriocins, including nisin produced by *Lactococcus lactis*, have demonstrated selective cytotoxic activity against human BC cell lines, such as MCF-7 [110]. Their anticancer activity is thought to involve apoptosis induction, membrane depolarization, increased membrane permeability, and modulation of immune-related pathways [111]. However, despite these promising findings, the clinical translation of bacteriocins remains limited by challenges related to stability, targeted delivery, and specificity [111,112].

Although these categories collectively illustrate the remarkable diversity of postbiotic preparations, it is important to recognize that the strength of the available evidence is not uniform. Soluble microbial metabolites, particularly SCFAs, are currently supported by the most extensive mechanistic and preclinical evidence, whereas structural macromolecules, extracellular vesicles, nanoparticles, and inactivated microbial cells remain comparatively less investigated, particularly in the context of BC. Consequently, the biological relevance and translational potential of these different postbiotic categories should be interpreted according to the current level of evidence available for each class. Overall, the current literature should not be interpreted as providing equivalent levels of evidence across all postbiotic categories. Rather, the field is characterized by varying degrees of mechanistic understanding and preclinical validation, highlighting important priorities for future investigation.

## 7. From Bench to Bedside: Current Challenges and Future Perspectives

Currently, 43 registered clinical trials are investigating postbiotic-based interventions in humans (Table 3), predominantly in non-oncological settings, including metabolic, gastrointestinal and inflammatory disorders, whereas their application in oncology remains extremely limited. To date, only one registered clinical trial (NCT07050940) is evaluating a postbiotic as an adjunct to anti-PD-1 therapy in advanced or metastatic melanoma, and no clinical studies have investigated postbiotics in BC, either as therapeutic agents or supportive interventions. Furthermore, although several registered studies have completed recruitment or are no longer actively recruiting, their results have not yet been published, further limiting the current clinical evidence.

This marked discrepancy between promising preclinical findings and clinical investigation highlights the limited translational readiness of postbiotics in oncology. Although numerous in vitro and animal studies have demonstrated anti-proliferative, pro-apoptotic, and immunomodulatory effects, these findings have yet to be validated in clinical settings that adequately reflect the complexity of the TME and host–microbiome interactions [8]. Several challenges currently hinder clinical translation, including the lack of standardized postbiotic formulations, batch-to-batch variability arising from differences in microbial strains and manufacturing processes, undefined dose–response relationships and pharmacokinetic profiles, and the absence of validated biomarkers of exposure and therapeutic response [113]. In addition, although postbiotics are generally considered safer than live probiotics, their immunomodulatory properties require careful evaluation in cancer patients receiving multimodal therapies, particularly immunotherapy. Regulatory uncertainty regarding their classification as dietary supplements, functional foods, or therapeutic agents further complicates product development and approval.

Beyond their potential direct anticancer activity, postbiotics may also contribute to preserving microbiome homeostasis during anticancer treatment. Chemotherapy, endocrine therapy, targeted therapies, and immunotherapy can profoundly disrupt gut microbial composition, promoting dysbiosis, gastrointestinal toxicity, impaired epithelial barrier function, and altered immune responses [73,114]. Microbial metabolism has also been shown to influence drug bioavailability and toxicity, for example through bacterial β-glucuronidase-mediated reactivation of drug metabolites [115] or depletion of butyrate-producing bacteria, which exacerbates mucosal injury [116]. Although these mechanisms have been extensively described in several malignancies, evidence specific to BC remains limited. Nevertheless, modulation of the gut microbiome through postbiotic interventions represents a promising strategy to improve treatment tolerability while potentially supporting antitumor immune responses, an area that warrants dedicated investigation.

Overall, the clinical application of postbiotics in BC remains at an early exploratory stage. Future studies should prioritize standardized formulations and rigorous pharmacological characterization. In addition, well-designed randomized clinical trials incorporating clinically meaningful oncological endpoints, together with integrated microbiome, metabolomic, and immune profiling, will be essential to identify predictive biomarkers, define the patients most likely to benefit, and determine whether postbiotics can be effectively integrated into precision breast cancer management.

## 8. Positive Microbiome-Mediated Enhancement of Therapeutic Efficacy

Beyond toxicity modulation, microbiomes also play a crucial role in shaping therapeutic responsiveness in BC. A balanced and functionally diverse microbial community appears to enhance the efficacy of multiple treatment modalities, including chemotherapy, immunotherapy, and endocrine therapy. Beneficial microbes can improve drug metabolism, promote antitumor immunity, and modify tumor cell signaling pathways that influence treatment sensitivity [43].

Preclinical studies demonstrate that specific bacterial taxa can enhance the cytotoxic effects of chemotherapeutic agents by modulating immune activation, enhancing reactive oxygen species generation, or increasing drug uptake within tumor tissues. In models of hormone receptor-positive BC, microbial metabolites—especially SCFAs and indole derivatives—modulate estrogen signaling and may improve response to endocrine therapies such as tamoxifen or aromatase inhibitors [117]. Conversely, decreased production of SCFAs, such as butyrate and propionate, has been associated with complications during chemo- and radiotherapy, highlighting the importance of a functional microbial community in supporting treatment efficacy. Additionally, gut microbial diversity has been linked to improved outcomes in patients receiving immune checkpoint inhibitors, owing to enhanced antigen presentation, increased CD8^+^ T-cell activity, and reduced immunosuppressive cell populations [117].

Emerging evidence also suggests that microbiome-targeted interventions, including probiotics, postbiotics, and dietary modulation, can synergize with conventional therapies to amplify their antitumor effects. Furthermore, microbial metabolites have shown potential to enhance the efficacy of immunomodulatory agents, suggesting a direct link between microbiome composition and therapeutic responsiveness [9]. Although most of these findings come from animal models, early-phase clinical studies in oncology suggest that adjusting microbial composition may represent a promising strategy to optimize therapeutic efficacy, though BC-specific evidence remains limited [43].

As the field advances, integrating microbiome assessment into BC management may enable personalized therapeutic approaches, the strategic use of commensal bacteria and their metabolites to enhance the efficacy of existing therapies, and improved predictions of treatment response through innovative adjunctive strategies designed to potentiate drug activity [9].

## 9. Conclusions

Growing evidence indicates that the human microbiome is a fundamental regulator of BC initiation, progression, and therapeutic response. Through complex metabolic, immunological, and endocrine interactions, microbial communities influence multiple hallmarks of cancer, including cell proliferation, inflammation, immune evasion, metastasis, and remodeling of the TME [5,41]. Within this framework, postbiotics—defined as non-viable microbial components and/or microbial-derived metabolites—have emerged as promising candidates for microbiome-based cancer interventions, offering the possibility of exploiting microbiome-associated biological activities without the safety concerns related to the administration of live microorganisms [12,17].

This review highlights that postbiotics constitute a highly heterogeneous class of bioactive compounds, including SCFAs, conjugated fatty acids, secondary BAs, indole derivatives, polyamines, bacteriocins, extracellular vesicles, structural microbial molecules, and EPS [46,49]. Collectively, these compounds exert pleiotropic anticancer effects by modulating epigenetic regulation; oncogenic signaling pathways, such as PI3K/AKT, NF-κB, and STAT3; inflammatory cytokine networks; oxidative stress responses; and immune surveillance mechanisms. In BC models, postbiotic interventions have been associated with reduced tumor cell proliferation, induction of apoptosis, inhibition of migration and metastatic potential modulation of the TME, and enhancement of immune recognition, with effects that may vary according to tumor molecular subtype and biological context [17,28].

Compared with live probiotics, postbiotics present several potential translational advantages, including improved safety, stability, manufacturability, and standardization, which are particularly relevant in oncology settings where patients may be immunocompromised or exposed to intensive therapeutic regimens [78,81]. However, despite the reduced risk associated with the absence of viable microorganisms, the safety profile of postbiotic interventions in cancer patients remains insufficiently characterized. The complex clinical and biological status of oncology patients, characterized by immune dysfunction, altered epithelial barrier integrity, and ongoing anticancer therapies, requires careful evaluation of tolerability, potential interactions with anticancer treatments, and possible effects on therapeutic response, particularly in patients receiving immunotherapy [43,47,116].

Despite their considerable therapeutic potential, several critical challenges currently limit clinical translation. A major issue concerns the standardization and characterization of postbiotic preparations. Variability in microbial strains, cultivation conditions, processing methods, inactivation procedures, and extraction strategies can substantially influence the composition and biological activity of postbiotic products. Furthermore, the absence of universally accepted quality-control parameters and reference standards represent a major obstacle to reproducibility across studies [77,80].

A further unresolved dilemma concerns the precise specificity of biological responses mediated by postbiotics. While a wealth of evidence confirms the antineoplastic activity of complex postbiotic preparations, whether these outcomes are primarily dictated by discrete bioactive molecules or emerge from synergistic crosstalk between diverse microbial metabolites and structural elements remains poorly defined [63]. The inherent heterogeneity of postbiotics—comprising SCFAs, bioactive peptides, extracellular vesicles, and cell wall fragments—complicates the attribution of distinct biological effects to individual constituents [23,46]. Moreover, numerous pathways targeted by these preparations, such as the regulation of apoptosis, inflammatory cascades, oxidative balance, and immune recruitment, are also susceptible to modulation by unrelated bioactive agents [40,56]. Consequently, future investigations must transition from evaluating bulk preparations toward isolating the molecular drivers of anticancer activity. Defining specific cellular targets and determining whether these signals represent unique microbiome-derived mechanisms or merely overlap with established therapeutic pathways will be essential for their integration into precision oncology.

Beyond fundamental mechanistic insights, several critical research priorities must be pursued to facilitate clinical transition. Rigorous pharmacological investigations are essential to determine the bioavailability, metabolic fate, and precise tissue distribution of postbiotic elements, ensuring that therapeutic concentrations can be effectively attained [63]. Furthermore, the identification of predictive biomarkers—spanning microbial community structure, metabolomic signatures, and tumor-specific immune profiles—is necessary to guide patient stratification and precision-based applications [81,118]. Finally, the development of sophisticated experimental platforms, such as humanized murine models and patient-derived organoids, will be vital to accurately recapitulate the intricate crosstalk between the microbiome and the breast TME [68].

Ultimately, the successful translation of postbiotics into BC management will require a transition from empirical testing of complex microbial-derived preparations toward a mechanism-driven development of standardized, molecularly defined, and clinically validated postbiotic therapeutics. Integrating microbiology, metabolomics, immunology, oncology, and precision medicine approaches will be essential to determine whether postbiotics can be safely incorporated into personalized BC treatment strategies, either as standalone interventions or as adjuvants capable of enhancing anticancer efficacy while reducing therapy-related toxicity [17,43].

## Figures and Tables

**Table 2:** Reported microbiome alterations across BC subtypes.

Subtype	Reported Microbiome Features	Representative Taxa (Examples)	Putative Functional Associations
ER+	Relatively higher diversity in some cohorts; enrichment of bacteria involved in estrogen metabolism	*Clostridium* spp., *Bacteroides* spp., *Escherichia coli*	Contribution to the estrobolome and modulation of circulating estrogens, potentially influencing hormone-dependent tumor growth [75,77]
HER2+	Limited and heterogeneous data; some studies report increased abundance of proteobacteria	*Proteobacteria*, *Firmicutes* (variable)	Possible interaction with immune signaling and response to HER2-targeted therapies; evidence remains preliminary [79]
TNBC	Reduced microbial diversity; enrichment of pro-inflammatory and opportunistic taxa	*Fusobacterium nucleatum*, *Enterobacteriaceae*, *Staphylococcus* spp.	Association with inflammatory pathways, tumor aggressiveness, and immune modulation [78]

**Table 3:** Overview of registered clinical trials investigating postbiotic interventions in humans. This table summarizes registered clinical studies evaluating postbiotic interventions and related compounds across different health conditions. The included trials mainly address non-oncological settings, such as metabolic, gastrointestinal, dermatological, and immune-related disorders, and include both ongoing and completed interventional studies. Importantly, clinical investigation of postbiotics in oncology remains extremely limited, with only one registered cancer-related trial identified to date and no clinical studies specifically evaluating postbiotics in breast cancer. Columns include the NCT number (trial identifier), study title, recruitment status, targeted conditions, interventions, study sponsors, and study type.

	NCT Number	Study Title	Status	Conditions	Interventions	Sponsor	Study Type
1	NCT06911073	A Study to Evaluate a Postbiotic in Supporting Weight Loss and Metabolic Health	Recruiting	Obesity and Overweight	Dietary Supplement: Postbiotic; Dietary Supplement: Placebo	ResBiotic Nutrition, Inc.	Interventional
2	NCT06474247	Postbiotic as Adjunct Treatment for Vaginal Candidiasis	Recruiting	Vaginal Candidiasis	Device: Postbiotic; Device: Placebo	Min-Tze LIONG	Interventional
3	NCT07050940	Anti PD-1 Plus Postbiotic Versus Anti PD-1 Alone in Locally Advanced or Metastatic, Treatment naïve, Cutaneous Melanoma Study	Recruiting	Melanoma Metastatic	Dietary Supplement: Postbiotic; Drug: AntiPD1	Azienda Ospedaliera di Perugia	Interventional
4	NCT06448182	Effect of a Postbiotic Intake on Glucose Control and Microbiota Composition of Type 2 Diabetic Subjects: a Randomized Controlled Trial.	Recruiting	Diabetes Mellitus, Type 2	Dietary Supplement: Postbiotic; Dietary Supplement: Placebo	Clinica Universidad de Navarra, Universidad de Navarra	Interventional
5	NCT05562739	Pilot Study to Assess the Effect of a Postbiotic Blend on Moderate Self-reported Anxiety	Completed	Anxiety	Dietary Supplement: Postbiotic; Dietary Supplement: Placebo	The Archer-Daniels-Midland Company	Interventional
6	NCT06346847	Study to Assess Effects of Postbiotic vs Placebo in Participants With Diarrhea-predominant IBS	Active, not recruiting	Diarrhea-Predominant Irritable Bowel Syndrome	Dietary Supplement: Postbiotic; Dietary Supplement: Placebo	A-Mansia Biotech S.A.	Interventional
7	NCT05912699	A Study to Evaluate the Effect of SlimBiotics L. Fermentum K8 Postbiotic on Weight Management and Metabolic Health Outcomes	Unknown status	Metabolic Health; Weight Loss; Stress; Sleep; Healthy Eating	Other: Slimbiotics Postbiotic; Other: Placebo	Slimbiotics	Interventional
8	NCT05440630	Study of Efficacy of a Probiotic and Postbiotic in Overweight and Obese Individuals	Withdrawn	Obesity	Dietary Supplement: Probiotics; Postbiotic; Placebo	The Archer-Daniels-Midland Company	Interventional
9	NCT05428137	Study of Safety and Efficacy of a Probiotic and Postbiotic in Overweight Individuals	Recruiting	Obesity; Abdominal Obesity	Dietary Supplement: Probiotics; Postbiotic; Placebo	The Archer-Daniels-Midland Company	Interventional
10	NCT06309121	Effects of the Postbiotic Blend ABB C3 on Adiposity and Glucose Metabolism in Children and Adolescents	Recruiting	Obesity, Childhood and Adolescent	Dietary Supplement: Postbiotic ABB C3; Placebo; Follow-up Postbiotic ABB C3 (Optional); Postbiotic Active Lifestyle Blend	Fundació Sant Joan de Déu	Interventional
11	NCT06795425	A Randomized, Double-Blind Study to Assess the Effect of a Postbiotic on Oxidative Stress and Exercise Performance	Not yet recruiting	Oxidative Stress; Healthy; Exercise-induced Muscle Damage	Dietary Supplement:	Lindenwood University	Interventional
12	NCT07165431	Effect of a Postbiotic Supplementation in Overweight and Obese Subjects: A Randomized Controlled Trial (PARABIOTICS-2).	Not yet recruiting	Nutrition, Healthy; Weight Loss; Overweight and Obesity	Dietary Supplement: Postbiotic + Nutritional Recommendations; Placebo + Nutritional Recommendations	Clinica Universidad de Navarra, Universidad de Navarra	Interventional
13	NCT07110896	Study Evaluating the Effect of Humiome^®^ Post LB on Stress, Anxiety, and Cognition in Aging Adults	Not yet recruiting	Healthy Aging	Dietary Supplement: Humiome Post LB (Postbiotic); Placebo	DSM Nutritional Products, Inc.	Interventional
14	NCT06352697	Probiotic Lysate (Postbiotic and Metabiotic) Supplementation for Adults MASLD Patients (DELI_MASLD Study)	Completed	Metabolic Dysfunction Associated Steatotic Liver Disease; Steatotic Liver Disease; Hepatic Steatosis	Dietary Supplement: Probiotic Lysate (Postbiotic and Metabiotic); Placebo	Bogomolets National Medical University	Interventional
15	NCT05804422	Probiotic Lysate (Postbiotic and Metabiotic) Supplementation for Adults NAFLD Patients (DELI_NAFLD Study)	Completed	Non-Alcoholic Fatty Liver Disease; Hepatic Steatosis	Dietary Supplement: Probiotic Lysate (Postbiotic and Metabiotic); Dietary Supplement: Placebo	Bogomolets National Medical University	Interventional
16	NCT05770076	Probiotic Lysate (Postbiotic and Metabiotic) Supplementation for Type 2 Diabetes Patients (DELI_Diab Study)	Completed	Obesity; Obesity, Abdominal; Insulin Resistance	Dietary Supplement: Probiotic Lysate (Postbiotic and Metabiotic); Placebo	Bogomolets National Medical University	Interventional
17	NCT06834984	Ritual Synbiotic+, a Dietary Supplement Designed to Impact Gastrointestinal Health, Mood, and Behavior in Women	Recruiting	Gastrointestinal Disease Symptoms	Dietary Supplement: Prebiotic, Probiotic, Postbiotic Combination; Placebo	Colorado State University	Interventional
18	NCT05367427	Heat-treated Postbiotic Consumption in Healthy People With Mild to Moderate Gastrointestinal Symptoms	Completed	Healthy; Gastrointestinal Dysfunction	Dietary Supplement: Heat-Treated Postbiotic Bifidobacterium Longum Consumption; Placebo	Instituto de Investigación Hospital Universitario La Paz	Interventional
19	NCT06474728	Effects of an Emollient Containing Postbiotic Saccharomyces and Lactobacillus on Pediatric Atopic Dermatitis	Recruiting	Atopic Dermatitis; Eczema; Emollient	Other: Treatment with an Emollient Containing Postbiotic Saccharomyces and Lactobacillus	Children’s Hospital of Fudan University	Interventional
20	NCT05475314	Effect of Postbiotic Product on Colonic Barriers in IBS	Completed	IBS (Irritable Bowel Syndrome)	Dietary Supplement: ReFerm(R)	Nordisk Rebalance A/S	Interventional
21	NCT05984004	Safety Evaluation of Intranasal Use of DSM 32444 Postbiotic in Humans	Completed	Rhino Sinusitis	Drug: 0.9% NaCl Isotonic Saline Solution; Combination Product: Bacillus Subtilis DSM32444, Inactivated	Huro Biotech Joint Stock Company	Interventional
22	NCT06889779	Study Evaluating the Efficacy of Different Mixes of HMO-2FL + Humiome^®^ Post LB on IBS Gastrointestinal Symptoms	Recruiting	IBS (Irritable Bowel Syndrome)	Dietary Supplement: MIX 1; MIX 2; Placebo	DSM Nutritional Products, Inc.	Interventional
23	NCT04639492	Postbiotic MBS and Metformin Combination in Patients With T2DM	Completed	Type-II Diabetes	Dietary Supplement: MBS Oral Solution	Microbio Co Ltd.	Interventional
24	NCT06738420	Medical Nutrition Formulations Enriched With Postbiotics for the Management of Gastrointestinal Disorders	Completed	Diarrhea of Diverse Etiology Requiring Oral Rehydration Therapy; Diarrhea and Gastrointestinal Symptoms Secondary to Enteral Nutrition	Dietary Supplement: ABB S3; ABB C22, Placebo	AB Biotek	Interventional
25	NCT06052592	Metabolomic Profile in Breastfed Late Preterm Infants	Completed	Formula Milk; Breast Milk Collection; Postbiotic Supplemented Formula Milk	Other: Milk	Ospedale Buon Consiglio Fatebenefratelli	Observational
26	NCT05562752	Pilot Study to Assess the Effect of a Probiotic Blend on Moderate Self-reported Anxiety.	Completed	Anxiety	Dietary Supplement: Probiotic; Placebo	The Archer-Daniels-Midland Company	Interventional
27	NCT04324749	Healthy Prebiotic and Postbiotic Effects of Peanuts and Peanut Butter: College Intervention Trial	Completed	Microbiota; Cognitive Change	Other: Roasted Peanuts (Group A); Peanut Butter (Group B); Control (Group C)	University of Barcelona	Interventional
28	NCT05999955	Safety and Efficacy of DSM 32444 Postbiotic in the Treatment of Acute Rhinosinusitis	Completed	Rhinosinusitis; Rhinitis	Drug: Neomycin/ Dexameythasone/Xylometazoline; Bacillus Subtilis DSM32444	Huro Biotech Joint Stock Company	Interventional
29	NCT06293911	Clinical Comparison of a Postbiotic-gel With Placebo Gel for Gingival Inflammation in Patients With Down Syndrome	Completed	Down Syndrome; Gingival Bleeding	Other: Biorepair Plus Parodontgel Intensive; Placebo Gel	University of Pavia	Interventional
31	NCT06738433	Efficacy and Tolerability of ABB I5 Prebiotic and ABB C22 Postbiotics for the Management of Constipation and Gastrointestinal Well-being: a Pilot Trial	Completed	Constipation	Dietary Supplement: ABB i5; ABB C24	AB Biotek	Interventional
32	NCT06868771	Study on the Effectiveness of an Emollient Cream Containing Pre and Postbiotic and Niacinamide 4% in the Treatment of Skin Xerosis and Itching in Oncological Patients Treated with Anti-EGFR	Recruiting	Xerosis Cutis	Other: Topical Treatment with the Pre- and Postbiotic Study Preparation and Niacinamide 4% and One with the 10% Urea-Based Preparation	Societa Italiana di Dermatologia Medica, Chirurgica, Estetica e di Malattie Sessualmente Trasmesse	Interventional
33	NCT05738746	The Efficacy of Pasteurised Akkermansia Muciniphila in Healthy Medical Workers	Completed	Stress; Healthy; Dietary Supplement	Dietary Supplement: Placebo; Pasteurized Akkermansia Muciniphila	Pomeranian Medical University Szczecin	Interventional
34	NCT05056025	Study to Evaluate the Response to Supplementation With Postbiotics in Patients With Macular Degeneration.	Unknown status	AMD; Soft Drusen; Reticular Pseudodrusen	Dietary Supplement: Postbotics and Vitamins; Vitamins	Institut de la Macula y la Retina	Interventional
35	NCT06355089	Intestinal Microbiota: Immunity, Recovery and Metabolic Health	Completed	Healthy	Dietary Supplement: Placebo Comparator; Whey Protein Hydrolysate Milk Product; Postbiotic and Whey Protein Hydrolysate Milk Product	University of Turku	Interventional
36	NCT06514573	Butyrate Supplementation in Children With Autism Spectrum Disorder (ASD) and Functional Gastrointestinal Disorders	Recruiting	Autism Spectrum Disorder (ASD)	Dietary Supplement: Sodium Butyrate; Placebo (Cornstarch)	Istituto Superiore di Sanità	Interventional
37	NCT07016087	Therapeutic Efficacy of Cutaneous Application of Postbiotic N-(1-carbamoyl-2-phenyl-ethyl) Butyramide (FBA) in Pediatric Subjects Affected by Atopic Dermatitis	Recruiting	Atopic Dermatitis	Other: Cosmetic Formulation, Experimental; Cosmetic Formulation, Usual	Federico II University	Interventional
38	NCT07079748	Study of the Effects and Mechanisms of Yeast Postbiotics on Persistent Allergic Rhinitis Symptoms	Not yet recruiting	Allergic Rhinitis	Dietary Supplement: Yeast Postbiotics; Placebo	LanZhou University	Interventional
39	NCT04151823	Effect of POSTbiotics Supplementation on Microbiome in OBese Children: the POST-OB Study	Unknown status	Childhood Obesity	Drug: Vitamin D3; Immunofos; Promotion of physical activity	University of Milan	Interventional
40	NCT06963840	Effect of Theronomic Ova-All Care on Biomarkers of Ovarian Health in Adult Female With Polycystic Ovarian Syndrome	Active, not recruiting	Polycystic Ovarian Syndrome (PCOS)	Dietary Supplement: Theronomic Ova-All Care	Wellizen Australia	Interventional
41	NCT06937814	The Effect of Humiome^®^ Post LB on Gut COMFort in healthY Adult Volunteers	Recruiting	GI Issues; Bowel Movements; Gastrointestinal Symptoms	Dietary Supplement: Humiome^®^ Post LB; Placebo	DSM Nutritional Products, Inc.	Interventional
42	NCT07215676	A Trial of a Multi-Component Nutritional Supplement in Hydrogen-Dominant Small Intestinal Bacterial Overgrowth	Not yet recruiting	Small Intestinal Bacterial Overgrowth Syndrome (SIBO)	Dietary Supplement: AV1PD1A	National University of Natural Medicine	Interventional
43	NCT05819424	EpiCor Clinical Study on Rapid Immune Modulating Effects	Completed	Immune Surveillance	Dietary Supplement: Yeast Fermentate (EpiCor)	Cargill	Interventional

## Data Availability

No new data were created or analyzed in this study. Data sharing is not applicable to this article.

## Abbreviations

The following abbreviations are used in this manuscript:

BC	Breast Cancer
SCFAs	Short-Chain Fatty Acids
FFA	Free Fatty Acid
AGS	Adenocarcinoma Gastric Stomach
SMOX	Spermine Oxidase
ETBF	Enterotoxigenic *Bacteroides fragilis*
DDR	DNA Damage Response
HDAC	Histone Deacetylase
MMP	Matrix Metalloproteinase
PAMPs	Pathogen-Associated Molecular Patterns
PPRs	Pattern Recognition Receptors
TLRs	Toll-Like Receptors
LPS	Lipopolysaccharide
ROS	Reactive Oxygen Species
Tregs	Regulatory T Cells
TME	Tumor Microenvironment
ECM	Extracellular Matrix
CAFs	Cancer-Associated Fibroblasts
TAMs	Tumor-Associated Macrophages
CAAs	Cancer-Associated Adipocytes
TNBC	Triple-Negative BC
MAPK	Mitogen-Activated Protein Kinase
ER+	Estrogen Receptor-Positive
ER−	Estrogen Receptor-Negative
HER2+	Human Epidermal Growth Factor Receptor 2-Positive
PPAR	Peroxisome Proliferator-Activated Receptor
BAs	Bile Acids
LCA	Lithocholic Acid
DCA	Deoxycholic Acid
UDCA	Ursodeoxycholic Acid
ILA	Indole-3-Lactic
IPA	Indole-3-Propionic Acid
IAA	Indole Acetic Acid
IAld	Indole-3-Aldehyde
IPyA	Indole-3-Pyruvic Acid
EMT	Epithelial-to-Mesenchymal Transition
TAAR	Trace Amine-Associated Receptor
EPS	Exopolysaccharides
GPR41	Free Fatty Acid Receptor 3, FFAR3
GPR43	Free Fatty Acid Receptor 2, FFAR2
LTA	Lipoteichoic Acid
